# The Maintenance of Epigenetic States by p53: The Guardian of the Epigenome

**DOI:** 10.18632/oncotarget.780

**Published:** 2012-12-11

**Authors:** Arnold J. Levine, Benjamin Greenbaum

**Affiliations:** ^1^ Institute for Advanced Study, Einstein Road, Princeton, N.J

**Keywords:** TP53, virus, cancer, cell death, epigenetic stability

The functions of the p53 tumor suppressor gene and its protein are to ensure fidelity of cellular replicative processes so as to minimize mutational errors even in the presence of a wide variety of environmental stresses. Among the stresses p53 senses and responds to are DNA damage including telomere erosion, hypoxia, nutrient deprivation, errors in ribosomal biogenesis, abnormal chromosome segregation and even the mutational activation of several diverse oncogenes. Each of these stresses increases the rate of mutagenesis and the frequencies of cancers. When a cell senses a stress, it signals to the p53 protein using a wide variety of protein modifications (phosphorylation, acetylation, methylation), which help to increase the half-life of the p53 protein and activate it as a transcription factor. The p53 protein then rolls out a transcriptional program resulting in cell cycle arrest, senescence, or apoptosis, repairing or eliminating the cell depending upon the cell type, its state of transformation or other environmental factors. That this is an important process is demonstrated by the observations that the p53 gene is the most commonly mutated gene in human cancers and individuals who inherit mutations in this gene develop cancers at a young age [1].

Leonova and Gudkov and their colleagues have just published a paper [2] that adds a number of new observations to this picture. First, p53 keeps several families of repetitive DNA elements transcriptionally silent, presumably by binding to the chromatin associated with these sequences. Indeed, the treatment of cells without p53, but not cells with a wild-type p53, with 5-aza-2'-deoxycytidine, which blocks DNA methylation and alters the chromatin to make it more transcriptionally active, results in a large increase in the rate of transcription of these repetitive DNA elements in a cell. These repetitive RNA species form double-stranded RNAs and increase the frequency of the dinucleotide CpG in RNA species and both these events engage toll-like receptors in the cell and initiate cytokine production including type 1 interferon which can then lead to apoptotic death of the host cells. Both hypomethylation of repetitive cellular DNA and the transcription of repetitive DNA elements have been reported in pancreatic cancers and other carcinomas [3] and the survival of these cancer cells suggests that they have evolved a mechanism to escape p53-interferon mediated cell death. Among the striking observations made in this work is one that has a great deal of support from previous studies; i.e. the absence of wild-type p53 in a cell appears to permit changes in the epigenetic profile of the cell. In other words the p53 protein guards against epigenetic change.

Jaenisch and his colleagues [4] first demonstrated that the CRE mediated loss of the DNA methyltransferase gene (Dnmt1) in cells results in a p53 activated apoptosis after the cells undergo several divisions. This suggests that p53 senses epigenetic changes or their consequences and kills cells undergoing such changes. The global loss of imprinting can then lead to widespread tumorigenesis in adult mice [5]. Takahashi and Yamanaka [6] demonstrated that the addition of four transcription factors (c-myc, KLF-4, Oct 4, Sox-2) to fibroblasts in culture results in the epigenetic reprograming of these cells so as to form induced pluripotent stem cells (iPS cells). The process was inefficient (about 0.1% of the cells), took a long time (3 to 4 weeks) and the iPS cells produced tumors when implanted (because of the myc and KLF-4 oncogenes). One could increase the efficiency of this process (up to 80%) and decrease the time it takes to make iPS cells (5 to 6 days) by employing cells that were derived from p53 knock-out mice [7,8]. The presence of wild-type p53 decreases the efficiency and slows the rate of changes during epigenetic reprograming. Indeed p53 knockout mice can have birth defects and these mice have an altered epigenetic methylation of their DNA [8]. In human breast cancers [9] and prostate cancers [10] that contain p53 mutations the transcriptional profiles of those tumors closely resemble embryonic stem cells in contrast to tumors with a wild-type p53 protein. The evidence is accumulating that the p53 protein is the guardian of the genome, the guardian of the epigenome and the guardian of repeated DNA sequences keeping them silent and protecting the cell from “interferon death”

One might ask what is the role of the transcripts produced by repetitive DNA elements in the cell in initiating interferon or cytokine death. A number of RNA viruses produce double-stranded RNAs, which can engage Toll-like receptor-3 and initiate the transcription of interferon genes. However, there is a second trigger of Toll- like receptors 7 and 8 by single stranded RNAs some of which derive from DNA sequences with high CpG dinucleotide compositions. The removal of methylation from the CpG dinucleotides in DNA, permitting transcription of that DNA, is in itself a trigger for the Toll-like receptor 9 resulting in a cytokine storm. Influenza viruses that replicate in birds contain a much higher CpG dinucleotide content than the Influenza viruses that replicate in humans [11]. In 1918 a bird Influenza virus infected the human population with a very high CpG dinucleotide frequency. This was the very pathogenic strain that may well have initiated cytokine storms in the population and were highly lethal for their hosts. Indeed RNAs with high CpG dinucleotides have been shown to produce interferon and cytokines in cell culture [12] and the influenza strains that entered the human population in 1918 (the H1N1 strains) have lowered their CpG dinucleotide content over the past 100 years of replication in the human host [13]. Coincident with this change in dinucleotide frequency the pathogenicity of these viruses has declined in human populations. Toll-like receptor 7 appears to mediate this cytokine storm in response to a set of RNAs with high CpG dinucleotide frequencies [13].

Thus the mechanisms of interferon mediated cell death in the absence of p53, as described by Gudkov and his colleagues demonstrate a role for p53 in stabilizing the epigenetic state of a cell, preventing it from changing. Second, in the event p53 function is lost and there are changes in the epigenetic state there is a failsafe, interferon mediated cell death. This is similar to the loss of p53 in cancer cells increasing the concentrations of the DJ-1 protein that is thought to play a role in reducing reactive oxygen species, a function that wild-type p53 commonly is employed to regulate (the Redox potential of a cell) [14]. We are beginning to find selective back up functions for the loss of wild-type p53 in cancer cells. As cancers develop under high mutation rates these back up functions fail and the cancer cells evolve. We will need to understand these processes.
